# Effects of an amylopectin and chromium complex on the anabolic response to a suboptimal dose of whey protein

**DOI:** 10.1186/s12970-017-0163-1

**Published:** 2017-02-08

**Authors:** T. N. Ziegenfuss, H. L. Lopez, A. Kedia, S. M. Habowski, J. E. Sandrock, B. Raub, C. M. Kerksick, A. A. Ferrando

**Affiliations:** 1 The Center for Applied Health Sciences, Division of Sports Nutrition and Exercise Science, 4302 Allen Road, Suite 120, Stow, OH 44224 USA; 2 Exercise and Performance Nutrition Laboratory, School of Health Sciences, Lindenwood University, 209 S. Kingshighway St., Charles, MO 63301 USA; 3 The University of Arkansas for Medical Sciences, 4301 West Markham, Little Rock, AR 72205 USA

**Keywords:** Insulin, Chromium, Insulin sensitivity, Muscle protein synthesis, Amino acids

## Abstract

**Background:**

Previous research has demonstrated the permissive effect of insulin on muscle protein kinetics, and the enhanced insulin sensitizing effect of chromium. In the presence of adequate whole protein and/or essential amino acids (EAA), insulin has a stimulatory effect on muscle protein synthesis, whereas in conditions of lower blood EAA concentrations, insulin has an inhibitory effect on protein breakdown. In this study, we determined the effect of an amylopectin/chromium (ACr) complex on changes in plasma concentrations of EAA, insulin, glucose, and the fractional rate of muscle protein synthesis (FSR).

**Methods:**

Using a double-blind, cross-over design, ten subjects (six men, four women) consumed 6 g whey protein + 2 g of the amylopectin-chromium complex (WPACr) or 6 g whey protein (WP) after an overnight fast. FSR was measured using a primed, continuous infusion of ring-d_5_-phenylalanine with serial muscle biopsies performed at 2, 4, and 8 h. Plasma EAA and insulin were assayed by ion-exchange chromatography and ELISA, respectively. After the biopsy at 4 h, subjects ingested their respective supplement, completed eight sets of bilateral isotonic leg extensions at 80% of their estimated 1-RM, and a final biopsy was obtained 4 h later.

**Results:**

Both trials increased EAA similarly, with peak levels noted 30 min after ingestion. Insulin tended (*p* = 0.09) to be higher in the WPACr trial. Paired samples t-tests using baseline and 4-h post-ingestion FSR data separately for each group revealed significant increases in the WPACr group (+0.0197%/h, *p* = 0.0004) and no difference in the WP group (+0.01215%/hr, *p* = 0.23). Independent t-tests confirmed significant (*p* = 0.045) differences in post-treatment FSR between trials.

**Conclusions:**

These data indicate that the addition of ACr to a 6 g dose of whey protein (WPACr) increases the FSR response beyond what is seen with a suboptimal dose of whey protein alone.

## Background

The metabolism of muscle proteins operates in a continual flux whereby post-absorptive periods result in a dominance of muscle protein breakdown and net catabolism [1]. Alternatively, rates of muscle protein synthesis dominate after periods of feeding, particularly when those feedings include an adequate dose of the essential amino acids (EAA) [2–4]. In recent years, attempts to determine the optimal protein dose to maximize muscle protein synthesis have been undertaken. A number of studies have indicated a maximal anabolic response of muscle protein synthesis. Moore in 2009 first examined the differential ability of titrated doses of egg protein (0, 5, 10, 20 and 40 g) to stimulate muscle protein synthesis (MPS) rates and concluded that a 20-g dose resulted in a maximal response [5]. Yang and colleagues used identical whey protein doses as the Moore study in elderly men and found that after exercise a 40-g dose elicited a maximal response [6]. Wiitard and investigators examined progressive doses of whey protein (up 40 g) and reported that a 20-g dose yielded the most robust response [7]. More recently, MacNaughton and colleagues reported that a 40-g dose of whey protein was responsible for higher increases in MPS compared to a 20-g dose, independent of how much lean mass an individual possessed. Collectively, these data suggest that a relative dose of approximately 0.18–0.40 g of protein per kilogram of body mass acutely elicits a maximal MPS response in humans [8, 9], depending on age and the presence of an exercise stimulus.

The role of insulin in muscle protein metabolism continues to garner interest from researchers. In the presence of adequate whole protein and/or EAA, insulin has a stimulatory effect on MPS, whereas in conditions of lower blood EAA concentrations, insulin has an inhibitory effect on protein breakdown with minimal impact of rates of muscle protein synthesis [10]. Consequently, any insulinogenic nutrient, or those that can improve insulin signaling, when combined with varying doses of EAA, could theoretically impact muscle protein balance. Indeed, Churchward-Venne and colleagues determined that a combination of added leucine (an essential amino acid with known insulinogenic properties [11]) and a dose of whey protein isolate that was deemed suboptimal (6.25 g) was able to favorably instigate acute increases in MPS and that the measured response of this combination was quantitatively similar to a 25 g dose of whey protein isolate [12]. Notably, the term “suboptimal” was used because of previous research that demonstrated submaximal muscle protein synthesis responses when absolutes doses of 5–10 g of protein were consumed [5].

In terms of insulin action, the trace mineral chromium continues to be investigated for its ability to improve insulin resistance and enhance insulin sensitivity in cell culture [13, 14], animal models [15] and humans [16, 17]. Collectively, these studies appear to indicate that chromium availability favorably impacts carbohydrate and lipid metabolism as well as GLUT-4 translocation. Furthermore, chromium appears to increase insulin responsiveness via an AMPK mediated pathway [18] and can instigate favorable changes to the insulin receptor [14]. Evans reported that supplementation with chromium picolinate improved cholesterol and glucose levels in non-diabetic and diabetic adults and was also associated with significant losses of fat mass and increases in lean mass [16]. Similarly, Kaats used a double-blind, placebo-controlled study to demonstrate that daily supplementation with chromium could favorably improve body composition in exercising humans [17]. While these studies point towards the ability of chromium to favorably impact various metabolic parameters, much more work needs to be done to clarify the impact that chromium may have on skeletal muscle physiology, particularly in populations that have suboptimal insulin sensitivity and/or protein kinetics.

Healthy aging (in the absence of other comorbidities) presents with increased levels of anabolic resistance that result in aged individuals needing to ingest higher amounts of protein to achieve maximal stimulation of MPS and the promotion of a positive balance of muscle protein [6, 19]. In addition, studies have reported a lower intake of protein in the elderly [20] and a greater need for protein in the elderly [21]. When combined, these two factors result in a relative lack of optimal stimulation of MPS, which ultimately may be tied to the loss of skeletal muscle with aging [22–24]. Thus, nutritional strategies that may facilitate improvements in MPS with smaller doses of protein are of great interest to researchers and clinicians who work with these populations.

The purpose of this study was to examine potential differences in glucose, insulin, plasma amino acids, and muscle protein synthesis between a suboptimal dose of whey protein and a combination of chromium and amylopectin in combination with the same protein dose. It was hypothesized that ingestion of the chromium-containing product would improve insulin signaling and fractional synthesis rates of skeletal muscle proteins.

## Methods

### Experimental approach

This investigation was completed as a randomized, double-blind, single-dose, comparator-controlled crossover trial. Ten apparently healthy men (*n* = 6) and women (*n* = 4) between the ages of 22–34 years were pre-screened using health history questionnaires, vital signs, and blood work prior to being enrolled in the study. All subjects were required to report to the laboratory after observing an eight hour fast (including caffeine) with all testing sessions taking place at near identical times in the morning. Additionally, subjects were asked to avoid exercising for 72 h prior to each research visit. Research procedures included venous blood draws and vastus lateralis muscle biopsies during a primed, constant infusion of L-[ring-d_5_]-phenylalanine (Cambridge Isotope Laboratories, Andover, MA). The fractional rate of muscle protein synthesis (FSR) was measured using the stable isotope tracer incorporation technique from vastus lateralis muscle biopsies performed two, four, and eight hours after initiating stable isotope tracer infusion. Blood samples were collected at baseline (time 0) and over an eight-hour time period (240, 270, 300, 330, 360, 390, 420 and 480 min) to assess changes in amino acid concentrations. Similarly, glucose and insulin concentrations were analyzed in venous blood samples collected 240, 270, 300, 330, 360, 390 and 480 min after tracer infusion. A skeletal muscle biopsy was performed two and four hours after tracer initiation followed by a single dose of the assigned test product administered orally. Study participants then completed eight sets of bilateral isotonic leg extension resistance exercise at a load equivalent to approximately 80% of their estimated one-repetition maximum (1-RM). A third biopsy was obtained four hours after test product ingestion. A washout period of 5 to 7 days was utilized before each subject was crossed over to the opposite condition and scheduled to complete an identical testing session. The order in which test products were provided was counterbalanced to prevent any order effect.

### Study participants

Ten healthy male (*n* = 6) and female (*n* = 4) participants (mean ± SD: 26.6 ± 3.7 years, 175.5 ± 10.9 cm, 78.56 ± 17.4 kg) were recruited to participate in this study. All participants read and signed an IRB-approved informed consent to participate document prior to their participation in the study (Integreview, Austin, TX; approval date: January 13, 2015). All participants completed a medical history and were screened by a study physician and determined to be normotensive and euglycemic with normal fasting insulin and HOMA-IR values. Potential participants were excluded if they had a history of diabetes, smoking, malignancy in the previous 6 months or any other clinical condition that the researchers felt would compromise their safe participation. Individuals who recently lost more than ten pounds, had prior bariatric procedures or were diagnosed or being treated for any chronic inflammatory condition or disease (Lupus, HIV/AIDS, etc.) were also excluded. Participants were not allowed to be taking any form of chromium supplements or any other dietary ingredient deemed by the research team to affect insulin sensitivity or glucose tolerance. Participants must have been regularly consuming animal proteins and agreed to continue following their normal resistance training and protein/amino acid supplementation patterns. Finally, participants were also excluded if they had a known allergy to wheat proteins, amylopectin or chromium, were regularly using any form of corticosteroids, anabolic-androgenic steroids or were already participating in another research study.

### Adverse event monitoring

All study participants were required to record any adverse events throughout the entire study protocol. Participants were queried for symptoms during and after their completion of the study protocol to assess both the incidence and severity of adverse events according to CTCAE grading and MedDRA guidelines.

### Dietary and physical activity controls

All study participants were asked to maintain their current dietary and exercise/physical activity habits. Care was taken to control diet and physical activity levels 24 h prior to each experimental trial as all participants were required to complete a 24-h dietary recall prior to their initial experimental trial. A copy of this recall was made and all study participants were instructed to duplicate their dietary intake 24-h prior to their subsequent trial. As mentioned previously, all study participants were asked to refrain from exercise for 72 h prior to each visit and to fast for eight hours prior to testing. All dietary records were analyzed by the same research team member using the clinical edition of NutriBase IX (Phoenix, AZ).

### Subject preparation

Participants reported to the laboratory after an overnight fast, were asked to void prior, and then height (in bare feet) and body mass were determined using a SECA Medical Scale (model 767, Hanover, Maryland USA). An 18–22-gauge polyethylene catheter was inserted into each arm by a research nurse; one was placed in a distal vein for heated blood sampling, and another was placed in the forearm for infusion of the stable isotope tracers.

### Blood sampling

All blood samples were collected into lithium heparin tubes and centrifuged. Plasma samples were then aliquoted to minimize future freeze/thaw cycles and stored at −80° C until analyses. Plasma blood samples (5 ml) were collected at baseline (0 min) and after the beginning of isotope infusion (240, 270, 300, 330, 360, 390, 420 and 480 min) for analysis of amino acid concentrations and isotopic enrichment. Insulin and glucose concentrations in plasma were measured at 240, 270, 300, 330, 360, 390 and 480 min after baseline sampling.

### Amino acid (isotopic) tracer

After insertion of peripheral catheters, a primed (5.04 μmol/kg), continuous (0.084 μmol/kg/min) infusion of the stable isotope ring-d_5_-phenylalanine was initiated. Stable isotopes were obtained from Cambridge Isotope Laboratories (Andover, MA), compounded by a licensed pharmacy (Cantrell Pharmacy, Little Rock, AR) and tested for sterility and pyrogenicity prior to administration. Prior to infusion into the subject, the isotope solution was passed through a sterile 0.22 μm (Millipore) filter.

### Muscle biopsy procedure

Muscle biopsies from the vastus lateralis were performed two, four and eight hours after initiation of tracer infusion. After the biopsy at four hours, a single dose of WPACr or WP was administered orally under supervision. Muscle biopsies were performed under local anesthesia (using sterile 1% lidocaine, without epinephrine) for pain management. A 5 mm Bergström needle was advanced into the muscle through a small (~1 cm) incision. Immediately after applying suction, a sample of the muscle (approximately 100–120 mg) was removed with the needle. The sample was cleaned with sterile saline, trimmed of any visible connective tissue, blotted, and then cut into three equal portions (~30 – 40 mg). All three samples were immediately frozen in liquid nitrogen and stored at −80° C. One portion was utilized for determination of muscle protein synthesis, and the others were retained for backup analyses.

### Supplementation protocol

**Fig. 1:**
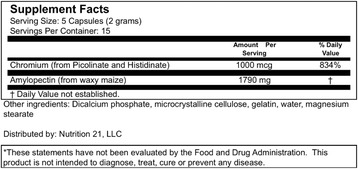
Supplements facts label for ACr

Upon consent, study participants were randomly assigned in a double-blind fashion to one of two trials: 6 g of whey protein isolate (BiPro USA, Eden Prairie, MN) + 2 g of the test product (Velositol™) or 6 g of whey protein isolate. All provided supplements were prepared in powdered form and packaged in coded generic containers for double-blind administration and dissolved in 8 oz of water immediately prior to oral dosing. All samples were blinded and matched for appearance, color, aroma and flavor by the study sponsor. Batch analysis of provided product at a third-party facility (Eurofins Scientific, Inc, Des Moines, IA USA, Certificate of Analysis # AR-15-QD-031109-01) was completed of both WPACr and WP and results indicated that levels of all bioactive ingredients were consistent with those reported on the Supplement Facts Label (see Fig. 1). After a 5–7 day washout, subjects crossed over and completed the opposite trial. The order in which test products were provided was counterbalanced to prevent any order effect.

### Resistance exercise protocol

As previously reported [25], all study participants then completed a single bout of bilateral leg extension exercise after supplement ingestion. Prior to beginning the study protocol all study participants determined their ten-repetition maximum and this load was used throughout the study. Each exercise trial consisted of eight sets of ten repetitions at their respective 10-repetition maximum load. A traditional plate-loaded leg extension machine was used and 90 s of rest was provided between each set. All repetitions were performed to near full-extension of the knee before returning to approximately 90–100° of knee flexion. Participants were instructed to extend the knee through the concentric phase for two seconds, briefly pause and return the knee eccentrically for a two second period. Each repetition was supervised by research personnel to ensure the proper load was used, each repetition was completed, and an appropriate range of motion and lifting cadence was followed. If a participant became too fatigued during the initial session to complete any repetition, the weight was lowered and this adjustment was matched during the subsequent visit. Thus, since all participants were required to complete the same number of repetitions at the same weight load, volume was equal between trials (within subjects).

### Calculation of fractional synthesis rates of muscle protein synthesis

Upon thawing, muscle tissues were weighed, and tissue proteins were precipitated with 0.5 ml of 4% SSA. The tissues were then homogenized and then centrifuged for collection of supernatant. The procedure was repeated two more times, and tissue intracellular free AAs were extracted from the pooled supernatant via the same cation exchange chromatography stated in plasma analyses and then dried under the Speed Vac. The remaining muscle pellet was washed, dried, and hydrolyzed in 0.5 ml of 6 N HCl at 105 °C for 24 h. Enrichments from muscle free and bound tracers were determined as in plasma analyses. Calculation of the fractional rate of muscle protein synthesis (FSR) was accomplished by the following equation:

FSR (%/hr) = [(E_p2_ – E_p1_)/(E_m_ X t)] X 60 X 100;

where E_P1_ and E_P2_ are the enrichments of bound l-[*ring*-^2^H_5_] phenylalanine in the first and second biopsies, respectively, and E_m_ is the calculated mean value of the enrichments of [*ring*-2H5] phenylalanine in the plasma pool. *t* is the time in minutes elapsed between the first and second muscle biopsy. Factors 60 and 100 were used to express FSR in percent per hour (Kim et al. 2014).

## Statistical analyses

A *p*-value of ≤0.05 was used to indicate statistical significance and values from 0.051 to 0.10 were deemed a trend. In all cases data are presented as means ± SD. All variables were tested for normality first using the Shapiro-Wilk test and followed up with individual skewness and kurtosis scores using 1.96 as a respective cut-off. Blood glucose, insulin and amino acid concentrations were compared using two-way factorial ANOVAs and t-tests when appropriate. Area under the curve (AUC) calculations were completed using the trapezoidal rule using Microsoft Excel (Seattle, WA). To investigate the presence of a gender effect due to our mixed gender cohort, a two-part approach was used. First, FSR data was analyzed using a univariate factorial ANOVA with gender and pre-treatment FSR as a covariate. Additionally, separate individual t-tests on both the pre-treatment and post-treatment FSR data for both conditions and with them pooled together. In no situation was gender found to operate as a significant confounder; consequently, all FSR data was analyzed as a mixed gender cohort. Muscle FSR values were then compared using ANCOVA (using the pre-treatment FSR value as the covariate). In addition, post-treatment FSR values and within-trial changes in FSR were compared using dependent t-tests. Effect sizes and 95% confidence intervals on the effect size were computed on the 4-h post-treatment FSR data. All statistical analysis and graphs were completed using IBM-SPSS for Windows, v21 (Armonk, NY) and Microsoft Excel (Seattle, WA).

## Results

### Compliance and adverse events

One female participant was initially removed after randomization due to dizziness that occurred after lidocaine injection and prior to the first muscle biopsy. This study participant was subsequently replaced by another eligible female. No mild, moderate or serious adverse events related to product ingestion were reported by any of the study participants.

### Dietary intake

Study participants were 100% compliant in completing dietary records as well as replicating their food and fluid intake as instructed prior to each testing condition. Independent t-tests revealed that energy (Male [*n* = 6]: 27.9 ± 5.9 kcal/kg/day vs. Female [*n* = 4]: 26.5 ± 7.3 kcal/kg/day, *p* = 0.74), carbohydrate (Male: 2.6 ± 0.8 g/kg/day vs. Female: 3.0 ± 0.6 g/kg/day, *p* = 0.49) and fat intake (2.3 ± 0.7 g/kg/day vs. 1.7 ± 0.3 g/kg/day, *p* = 0.14) normalized to body mas in kg was not different between genders. Protein intake was greater (*p* = 0.003) in females (1.7 ± 0.2 g/kg/day) than in males (1.2 ± 0.2 g/kg/day).

### Plasma amino acid responses

Total BCAA concentrations in both WPACr and WP peaked 30 min post-treatment (270 min time point) and remained elevated (*p* < 0.05) for another 30 min (300 min time point) before returning back to pre-treatment levels (Table 1). Two-way ANOVA revealed no trial x time interactions (*p* = 0.31) for changes in total branched-chain amino acids between the two conditions.

**Table 1:** Plasma concentrations (means ± SD) of leucine, isoleucine, valine and total BCAA for WPACr and WP across all time points

	Group	0 min	120 min	240 min	270 min	300 min	330 min	360 min	390 min	420 min	480 min
Leucine	WPACr	122 ± 24	115 ± 18	113 ± 16	216 ± 32^†^	163 ± 26^†^	133 ± 20	128 ± 19	124 ± 19	124 ± 17	128 ± 15
(μM)	WP	105 ± 25	101 ± 19	103 ± 24	207 ± 35^†^	163 ± 24^†^	132 ± 23^†^	124 ± 22^†^	119 ± 21^†^	118 ± 21^†^	122 ± 22^†^
Isoleucine	WPACr	65 ± 15	60 ± 9^†^	57 ± 9^†^	107 ± 14^†^	79 ± 12^†^	62 ± 9	59 ± 9	57 ± 9^†^	57 ± 8	60 ± 7
(μM)	WP	60 ± 13	56 ± 9^†^	57 ± 12^†^	110 ± 19^†^	84 ± 14^†^	67 ± 12	62 ± 12	60 ± 10	59 ± 10	62 ± 11
Valine	WPACr	216 ± 35	207 ± 30^†^	197 ± 27^†^	254 ± 38^†^	222 ± 29	199 ± 27^†^	197 ± 28^†^	191 ± 29^†^	188 ± 26^†^	196 ± 27^†^
(μM)	WP	208 ± 38	198 ± 32^†^	199 ± 33	261 ± 37^†^	228 ± 31	205 ± 34	200 ± 31	196 ± 31	195 ± 33^†^	199 ± 35
Total BCAAs	WPACr	404 ± 71	382 ± 56^†^	367 ± 50^†^	576 ± 81^†^	464 ± 64^†^	394 ± 53	383 ± 54	372 ± 55	369 ± 49	384 ± 48
(μM)	WP	387 ± 55	364 ± 45^†^	371 ± 58	572 ± 99^†^	479 ± 57^†^	411 ± 51	394 ± 50	387 ± 46	380 ± 50	392 ± 52

*WPACr* Whey protein + Amylopectin + Chromium, *WP* Whey protein, *μM* micromoles. ^†^ = Significantly different from 0 min (*p* < 0.05)

Individual serum concentrations of leucine, isoleucine and valine all followed a similar pattern of response with a significant increase (*p* < 0.05) occurring approximately 30 min after ingestion (270 min time point) and remained elevated for another 30 min (300 min time point). Two-way ANOVA revealed no trial x time interactions for leucine (*p* = 0.45), isoleucine (*p* = 0.51) and valine (*p* = 0.35) of these individual amino acids nor were pair-wise differences found to be statistically significant between trials at any time point. Amino acids responses are outlined in Table 1.

### Plasma glucose and insulin responses

Two-way mixed factorial ANOVA revealed no significant trial x time interaction (*p* = 0.22) for plasma glucose responses. Significant within-trial reductions in plasma glucose were seen in WPACr in all time points after the 300 min time point. Independent t-tests comparing the AUC for plasma glucose responses in the first two hours (240–360 min, *p* = 0.162) and four hours (240 – 480 min, *p* = 0.102) after test product administration were not significant. Plasma glucose responses are outlined in Table 2.

**Table 2:** Plasma concentrations (means ± SD) of glucose and insulin for WPACr and WP across all time points

	Group	120 min	240 min	270 min	300 min	330 min	360 min	390 min	480 min
Glucose	WPACr	5.37 ± 0.34	5.29 ± 0.26	5.27 ± 0.33	5.14 ± 0.23	5.14 ± 0.19	5.14 ± 0.25	5.04 ± 0.27	4.97 ± 0.24
(mM)	WP	5.00 ± 0.21	5.07 ± 0.45	5.17 ± 0.38	5.06 ± 0.27	5.08 ± 0.28	5.02 ± 0.31	4.92 ± 0.32	4.88 ± 0.28
Insulin	WPACr	5.61 ± 1.69	4.50 ± 1.48	12.74 ± 2.53^†^	6.87 ± 2.18^†^	4.50 ± 1.12	3.81 ± 1.15	3.49 ± 0.95^†^	3.84 ± 1.43
(mIU/mL)	WP	4.68 ± 2.28	4.42 ± 2.05	10.0 ± 4.32^†^	6.13 ± 2.62^†^	4.69 ± 1.83	4.22 ± 1.90	3.77 ± 1.43	3.33 ± 1.38

*WPACr* Whey protein + Amylopectin + Chromium, *WP* Whey protein, *μM* micromoles, *mIU/mL* milliinternational units per milliliter of blood, ^†^ significantly different than 240 min time point (*p* < 0.05)

Two-way mixed factorial ANOVA using plasma insulin responses revealed a trend (*p* = 0.09) for a trial x time interaction. Within-trial changes in comparison to the 240-min sample in both groups resulted in significant increases in plasma insulin concentrations (*p* < 0.05) at 270 and 300 min (30 and 60 min post-treatment, respectively). Independent t-tests comparing the AUC for plasma insulin responses in the first two hours (240–360 min, *p* = 0.346) and four hours (240 – 480 min, *p* = 0.478) after test product administration were not significant. Plasma insulin responses are outlined in Table 2.

### Muscle Fractional Synthesis Rate (FSR)

Independent t-tests were computed on both the pre-treatment (*p* = 0.74) and 4-h post-treatment (*p* = 0.76) FSR data for all conditions. In all instances, no significant differences were found between genders for either condition or when both condition were pooled at either time point.

**Fig. 2:**
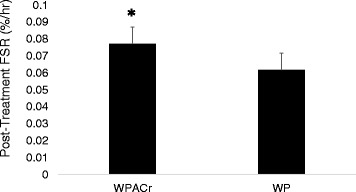
Mean ± SD post-treatment fractional synthesis rates using plasma precursor enrichment values for WPACr and WP 4 h after the treatment was administered. * = ANCOVA on 4-h post-treatment FSR value, *p* = 0.054. The within-trial change (4 h post-treatment FSR vs. baseline FSR data) for FSR was *p* = 0.0004 for WPACr vs. *p* = 0.23 for WP. In addition, independent t-test comparing post-treatment FSR between trials was *p* = 0.045. WPACr = Whey protein + Amylopectin + Chromium. WP = Whey protein

Pre-treatment FSR data was not significantly different between WP and WPACr (*p* = 0.78). At the 4-h post-treatment time point, WPACr yielded a more robust FSR response (i.e. 48% increase from baseline) compared to the Control trial (24% increase from baseline; Fig. 2). ANCOVA comparing the post-treatment FSR values (using the pre-treatment value as the co-variate) revealed a strong trend between trials (*p* = 0.054). Independent t-tests confirmed significant (*p* = 0.045) differences in post-treatment FSR between trials, as well as a statistically significant (within-trial) increase using paired samples t-test during WPACr (48%, *p* = 0.0004) vs. a non-significant increase during the WP (24%, *p* = 0.23). The average effect size for the 4-h post-treatment data was 0.93 (95% CI: 0.00–1.85), indicating a large treatment effect during the WPACr trial.

## Discussion

The primary finding of the present study is that despite similar changes in plasma EAA responses, adding a novel amylopectin/chromium-containing complex to a suboptimal dose of whey protein magnified the increase in MPS from protein intake and resistance exercise (assessed four hours post-ingestion). A key strength of our study design is the randomized, counter-balanced, within-subject crossover approach we used to examine the potential differences between the two experimental conditions.

Of note, the MPS response with WPACr was approximately two times greater than the response seen in the whey protein only condition (i.e. 48% vs. 24% increase from baseline, Fig. 2). Mechanistically, amino acid levels significantly increased in both conditions, suggesting that the amount of substrate available for new muscle proteins to be resynthesized was not favorably tilted towards the WPACr trial. While it is tempting to speculate that the ACr complex afforded a more favorable biochemical environment upon which new proteins could be synthesized, this assertion is premature given our current study design. Future work examining the expression of various intramuscular signaling proteins (i.e., mTOR, p70s6k, etc.) is needed to explore this possibility.

To our knowledge, these results are among the first to illustrate the impact of a novel amylopectin chromium-containing complex on the stimulation of mixed muscle protein synthesis. In seeking an explanation for our study outcomes, the purported ability of chromium to favorably alter insulin metabolism [14, 26, 27] is an important mechanistic consideration. This suggestion is supported by previous cell culture [13, 14], animal [15] and human work [16, 17] that has indicated chromium picolinate can improve carbohydrate and lipid metabolism, GLUT-4 translocation and others aspects of insulin metabolism. In this respect, Evans and Bowman reported that chromium picolinate can increase the internalization of insulin and markedly increase leucine uptake in cultured rat skeletal muscle cells [13]. Other cell culture work by Wang and colleagues reported that treatment of chromium in cultured human cells led to greater activation of insulin receptor kinase activity [14]. Cefalu used an animal model and concluded that oral chromium treatment significantly increased glucose and insulin areas under the curves as well as improved GLUT-4 metabolism leading them to conclude that chromium picolinate supplementation enhances insulin sensitivity and glucose disappearance [15]. Finally and in a series of human studies, Evans reported that 200 micrograms of chromium picolinate improved cholesterol and glucose levels in non-diabetic and diabetic adults, while two other studies in young men who were resistance training experienced significant losses of body fat and increases in lean mass [16]. Additional human work published in 1998 used a double-blind, placebo-controlled approach and also concluded that daily supplementation with chromium significantly improves multiple body composition parameters [17].

While the exact role(s) of insulin in muscle protein metabolism continues to be clarified, insulin has a demonstrated stimulatory effect on muscle protein synthesis when adequate EAA precursors are present, and seems to work more towards reducing muscle protein breakdown when EAA concentrations are reduced [10]. In this respect, investigating the post insulin receptor signal transduction pathways and phosphorylation cascades, including activation of IRS-1 (insulin receptor substrate-1) /PI3K (phosphatidylinositol-3 kinase)/Akt (protein kinase B)/mTORC /p70S6 kinase axis, are central to understanding the molecular mechanisms of muscle protein synthesis [28–30]. If WPACr acutely enhances these intracellular responses to insulin as indicated by previous work in culture [18], animal [13] and human studies [16], then it may potentially augment the anabolic response of skeletal muscle to an otherwise suboptimal dose of whey protein. This is an important consideration as the present study examined acute changes in fractional synthesis rates of mixed muscle proteins, but did not explore the impact of the chromium-containing compound on overall muscle protein balance, rates of muscle protein breakdown, or whole-body net protein balance. It is also worth mentioning that an interaction between the whey protein and amylopectin could have also impacted the observed changes in muscle FSR, however, this interaction is deemed minimal. The inclusion of amylopectin was primarily from a formulary perspective to operate as a transport vehicle; further, the provided dosage (~2 g) has not been shown to exhibit a substantive physiological impact.

While our four-hour post-treatment FSR changes provide encouraging preliminary evidence that the chromium-containing complex may potentiate the anabolic response seen in a mixed muscle sample after a resistance training stimulus, these conclusions have potential limitations (e.g., small sample size and our mixed gender cohort). In this respect, our sample size is quite consistent with previous studies that have employed similar study designs using identical methodologies that are known to have excellent sensitivity for detecting changes in FSR [2–4]. In addition, any impact of gender was deemed minimal because our independent t-tests between genders on all FSR data revealed no instance where gender differences were present, as did univariate factorial ANOVA with gender as a covariate. These findings support previous work by Markofski that demonstrated no difference in basal rates of MPS between genders [31]. In addition, it is acknowledged that our muscle biopsy samples were analyzed as a mixed muscle sample and thus the observed effects may or may not be specific to myofibrillar protein synthesis.

The fitness and athletic communities could potentially benefit from our findings through identification of means to drive muscle anabolism while reducing the overall daily caloric load. Additionally, the aging and insulin resistant populations are particularly intriguing candidates for translation of this line of research into practice. In particular, the aged have previously been shown to exhibit a certain level of anabolic resistance to the stimulatory effect of amino acids [19] resulting in larger doses of the essential amino acids and intact proteins required to stimulate maximal rates of muscle protein synthesis [6]. This is problematic given evidence suggesting that protein intake in the elderly is reduced [20].

## Conclusions

In conclusion, this study demonstrates that the addition of the amylopectin/chromium-containing complex to a suboptimal dose of whey protein [12] improves the muscle anabolism response to acute resistance exercise beyond that of the protein dose alone in young, healthy subjects. Future research should confirm these data and seek to better understand the mechanisms responsible for the observed results.
